# Human infection with *Dicrocoelium dendriticum* in Turkey

**DOI:** 10.4103/0256-4947.60525

**Published:** 2010

**Authors:** Zeynep Taş Cengiz, Hasan Yilmaz, Ahmet Cumhur Dülger, Mutalip Çiçek

**Affiliations:** a From the Department of Parasitology, Medical Faculty of Yüzüncü Yil University, Van, Turkey; b From the Department of Internal Medicine, Medical Faculty of Yüzüncü Yil University, Van, Turkey; c From the Department of Microbiology, Medical Faculty of Dicle University, Diyarbakir, Turkey

## Abstract

Human dicrocoeliosis is reported sporadically in various parts of the world. We report a case in a 21-year-old male, who had right upper abdominal pain, weight loss, and chronic relapsing watery diarrhea three to four times daily for four weeks. The patient had abdominal tenderness to palpation in the right upper quadrant. Alkaline phosphatase, alanine aminotransferase, and serum immunoglobulin E levels were slightly elevated; all other biochemical and hematological findings were in their normal ranges. The duodenal biopsy samples were normal and an abdominal ultrasonography showed no biliary or hepatic abnormality. Stool microscopy revealed numerous eggs of *Dicrocoelium dendriticum.* As pseudoparasitosis can result from eating raw, infected animal liver, the patient was given a liver-free diet for three days, to rule out that possibility. Subsequent stool examinations showed eggs in each of the samples indicating that the infection was genuine. The patient was treated with triclabendazole 10 mg/kg in a single dose. Four weeks later, no parasite eggs were detected in the microscopic examination of the stool samples. The patient got better gradually and the symptoms disappeared. Physicians should keep in mind parasitic diseases such as the rarely encountered dicrocoeliosis.

Dicrocoeliosis is caused by *Dicrocoelium dendriticum*, a 5 to 15 mm long and 1.5 to 2.5 mm wide parasitic fluke that usually infests the gall bladder and bile ducts of herbivores, such as, sheep, goat, cattle, and sometimes humans. The infection occurs as a result of oral ingestion of ants (belonging to the genus *Formica,* which is the second intermediate host) infected with metacercaria, via food.^1^–^3^ Although spurious infections are sometimes encountered in stool examinations because of the ingestion of infected liver, real infections with this parasite are rarely reported in Turkey or elsewhere in the world in humans.^1^–^4^ The infection is known to be widespread in ruminants in Turkey.^3^

Diarrhea lasting longer than four weeks warrants evaluation, to exclude a serious underlying pathology. In contrast to acute diarrhea, most of the causes of chronic diarrhea are noninfectious. However, some parasitic diseases may cause chronic diarrhea.^5^ Although *D. dendriticum* is less pathogenic than *Fasciola hepatica*, it leads to extensive damage in the liver by penetrating even to the most slender bile ducts. Accordingly, symptoms such as irritation in bile ducts, infectious hepatitis, a swollen abdomen, enlarged and painful liver, diarrhea, constipation, eosinophilia, and anemia are the symptoms also seen in fascioliosis.^1^–^3^

The prevalence of *D. dendriticum* in our region is unknown. People living in the provinces generally only visit the hospital in cases of emergency. If an emergency occurs they prefer to first go to a small health center without equipment, where parasitological examinations are not usually available. This case is important because it attracts attention to parasitic diseases such as dicrocoeliosis, which are non-endemic and cannot be easily diagnosed clinically.

## CASE

**Figure 1:**
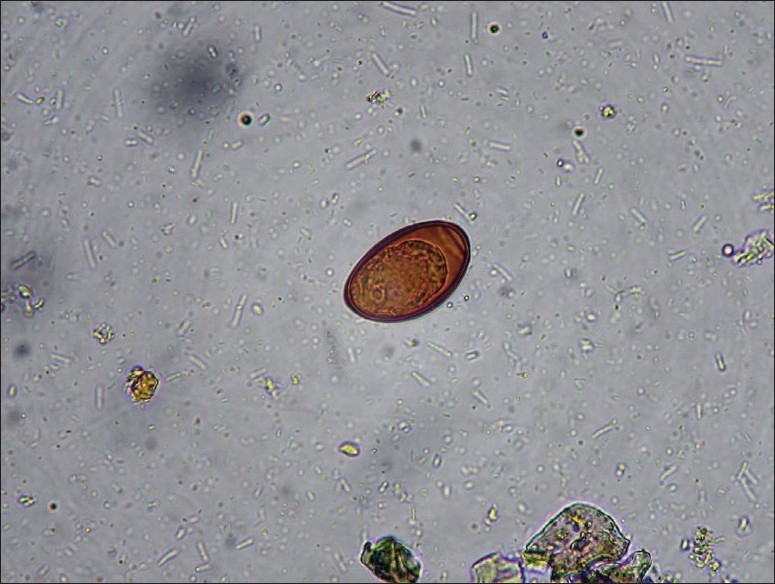
*D. dendriticum* egg (42 μm × 26 μm) detected in the stool microscopy of the patient.

Our patient was a 21-year-old male who had right upper abdominal pain, weight loss, and chronic relapsing watery diarrhea three to four times daily for four weeks. On physical examination, the patient had abdominal tenderness to palpation in the right upper quadrant. There was no eosinophilia, and the erythrocyte sedimentation rate (ESR) was 14 mm/h. Alkaline phosphatase (ALP), alanine aminotransferase (ALT), and serum immunoglobulin E levels were slightly elevated, (367 U/L (normal reference range, 0-270 U/L), 41 U/L (normal reference range, 0-41 U/L), and 253 U/mL (normal reference range, 0-100 U/mL), respectively. Direct and indirect bilirubin levels were in the normal range. Serologic tests for celiac disease were negative. There were no hormonal abnormalities causing watery diarrhea. Hepatitis B surface antigen was positive, but the HBV DNA level was low and the antibody to the hepatitis delta virus was negative. Furthermore, there were no signs of hepatic disease due to hepatitis B. All other biochemical and hematological findings were in the normal range. The duodenal biopsy samples were normal and the abdominal ultrasonography showed no bile duct or hepatic abnormalities. Stool microscopy of the patient revealed numerous eggs of *D. dendriticum* (Figure 1), which were asymmetric, dark-brown, and had an operculated shell by wet smear in 0.9% saline solution and by the flotation method in saturated zinc sulfate. To confirm the result of the previous stool examination, new examinations were repeated consecutively over the next three days, after placing the patient on a liver-free diet, to determine whether the infection was real or psuedoparasitosis.

The eggs were seen in each of the samples, which indicated that the infection was genuine and not a spurious infection. In addition, it was also discovered that the patient had not eaten liver or liver products for the last seven days before the stool examinations were performed.

Following confirmation of the diagnosis, the patient was treated with triclabendazole (Fasinex^®^; Ciba-Geigy, Switzerland) 10 mg/kg in a single dose. Four weeks after treatment, stool samples of the patient were examined consecutively for three days using the same methods. The patient was almost free of symptoms and stool examinations for the parasite remained negative. The patient was completely treated with a single dose of the anthelmintic.

## DISCUSSION

There are many causes of chronic diarrhea in humans.^5^ Liver flukes are one of the causes of infection for chronic diarrhea.^1^,^2^,^4^ Only a few dicroceliasis cases have been found among people in Turkey.^4^,^6^–^13^ In the Cerrahpaşa Medicine Faculty Hospital (Istanbul University), dicroceliasis was detected in a 65-year-old female patient. The bile duct of the patient was obstructed with *D. dendriticum*, and the patient was successfully treated with a single dose of triclabendazole (10 mg/kg).^12^ At Kırıkkale University, surgeons operated on a 12-year-old girl with cholelithiasis due to *D. dendriticum*.^13^

Cases of human dicrocoeliosis have been reported sporadically in various parts of the world.^14^–^18^ In a study in Saudi Arabia, *D. dendriticum* eggs were detected in 208 patients from 1984 to 1986, and at least seven of the 208 patients were diagnosed as having a real infection.^14^ In another study in Saudi Arabia, true dicroceliasis was detected in 32 of 1196 patients by re-examination after three days of a liver-free diet.^15^ In Germany, dicroceliasis was reported in a 36-year-old patient, who was successfully treated with triclabendazole (700 mg, single dose).^16^ Another case of dicroceliasis reported in Germany, was of a 21-year old Afghani woman who had traveled to Germany as an immigrant.^17^ In Czechoslovakia, the first case was reported in an 11-year-old boy in 1989.^18^

Although the parasite eggs have been detected high rates in stool samples in some studies in Turkey and around the world,^6^–^11^,^14^,^15^ the researchers have not always clearly indicated whether infections were real; in some cases they may have been spurious. However, because experienced parasitologists are unavailable in some health centers, we think that some dicrocoeliasis cases may be overlooked.

The clinical symptoms of dicroceliasis are almost always the same as in fascioliosis. Although sometimes no symptoms are seen in patients infected with *D. dendriticum*, acute clinical symptoms such as eosinophilia, abdominal distention, painful liver, right upper abdominal pain, diarrhea, constipation, and anemia are observed in severely infected cases.^1^ Our patient had only weight loss, watery diarrhea three to four times a day, and right upper abdominal pain for four weeks. Although his immunoglobulin E level was high, no eosinophilia or organomegaly was seen.

Triclabendazole is very effective in a single dose of 10 mg/kg, especially in the treatment of fasciolosis.^19^–^22^ Therefore, we decided to use triclabendazole (Fasinex^®^; Ciba-Geigy) 10 mg/kg in a single dose for our patient. Following the treatment, stool examinations were performed by using the wet smear and flotation methods, for three consecutive days. No parasite eggs were detected in the stool samples, and the patient started to get better gradually and the symptoms began to disappear.

The real prevalence of *D. dendriticum* in our region is unknown because people only go to the hospital when there is an emergency. If an emergency occurs they prefer to first visit village clinics, and often these clinics cannot perform parasitological examinations.

In conclusion, dicroceliasis can be associated with chronic watery diarrhea and right upper abdominal pain, and stool samples of the patients must be examined for intestinal parasitic diseases in health centers. Physicians should keep in mind parasitic diseases, even though they are encountered only rarely.
